# Organerhaltende Therapie des muskelinvasiven Blasenkarzinoms: 10-Jahres-Daten der britischen BC2001-Studie

**DOI:** 10.1007/s00066-023-02052-2

**Published:** 2023-02-23

**Authors:** Felix Grabenbauer, Michael Flentje

**Affiliations:** Klinik und Poliklinik für Strahlentherapie, Uniklinikum Würzburg, Würzburg, Deutschland

## Hintergrund

BC2001, die größte randomisierte Studie zur organerhaltenden Behandlung des muskelinvasiven Blasenkarzinoms (MIBC), zeigte eine Verbesserung in der lokoregionären Tumorkontrolle durch Hinzunahme von 5‑Fluorouracil (5-FU) und Mitomycin C (MMC) zur Bestrahlung. Die 5‑Jahres-Daten wurden bereits 2012 im N Engl J Med publiziert [1]. Die aktuelle Publikation betrifft Langzeitdaten mit 10-Jahres-Follow-up [2].

## Material und Methoden

Angelegt wurde die randomisierte, kontrollierte Phase-III-Studie im multifaktoriellen 2 × 2-Aufbau, d. h., das Patientenkollektiv wurde zu zwei Fragestellungen jeweils zweiarmig randomisiert. Einerseits erfolgte die Randomisierung in die zwei Arme Radiochemotherapie vs. alleinige Bestrahlung. Die zweite Randomisierung erfolgte anhand der Bestrahlungsvolumina in Standardbestrahlung des gesamten Blasenvolumens vs. hoch dosierte Teilvolumenbestrahlung der Blase; restliche, tumorferne (> 1,5 cm) Blasenabschnitte wurden dabei mit 80 % der Referenzdosis bestrahlt. Die Bestrahlung erfolgte entweder moderat-hypofraktioniert mit 55 Gy in 20 Fraktionen (ED 2,75 Gy) oder konventionell-fraktioniert mit 64 Gy in 32 Fraktionen (ED 2 Gy). Die simultane Chemotherapie bestand aus 5‑FU (500 mg/m^2^ KOF d1‑5, 16–20, i.v., kontinuierliche Infusion) und Mitomycin C (12 mg/m^2^ KOF d1, Bolus i.v.). Endpunkte waren die lokoregionäre Kontrolle (primärer Endpunkt), invasive lokoregionäre Kontrolle (primärer Endpunkt für Randomisierung Radiotherapie), Rate an Salvage-Zystektomien, progressionsfreies Überleben (PFS), krankheitsfreies Überleben (DFS), metastasenfreies Überleben (MFS), blasenkrebsspezifisches Überleben (BCSS), Gesamtüberleben und Toxizität. Das mediane Follow-up betrug 9,9 Jahre.

## Ergebnisse

Die Radiochemotherapie verbesserte gegenüber der alleinigen Bestrahlung sowohl die lokoregionäre Kontrolle (HR 0,61, *p* = 0,004) und die invasive lokoregionäre Kontrolle (HR 0,55, *p* = 0,006) als auch das PFS (63 % vs. 49 %, *p* = 0,004). Ein Trend war erkennbar für das DFS (HR 0,78, *p* = 0,069) und das MFS (HR 0,78, *p* = 0,089), jedoch nicht für das Gesamtüberleben (HR 0,88, *p* = 0,3). Die 5‑Jahres-Zystektomierate betrug 14 % bei Radiochemotherapie gegenüber 22 % bei Strahlentherapie allein (HR 0,54, *p* = 0,034). Zwischen Standard- und hoch dosierter Teilvolumenbestrahlung ergaben sich keine Unterschiede.

## Schlussfolgerung der Autoren

Die BC2001-Studie zeigt einen Vorteil der Radiochemotherapie gegenüber der alleinigen Radiotherapie, der sich auch im Langzeitverlauf bestätigt.

## Kommentar

Die Zystektomie galt und gilt immer noch als Standardtherapie des muskelinvasiven Blasenkarzinoms [3]. Allerdings betrug die Rate einer kompletten Kontinenz selbst bei orthotoper Neoblase an spezialisierten Zentren nur zwischen 70 und 93 % und bei männlichen Patienten konnte die erektile Funktion nur in 33–100 % erhalten werden [4, 5]. Die 5‑Jahres-Überlebenszeiten nach Zystektomie liegen zwischen 40 und 60 %.

Die organerhaltende trimodale Behandlung des muskelinvasiven Blasenkarzinoms (TUR plus RT plus simultane Chemotherapie) steht den Resultaten der radikalen Zystektomie in Bezug auf das Überleben keineswegs nach [6, 7]. Es gibt zwar weiterhin keine randomisierten Studien mit direktem Vergleich der Verfahren, aber eine kürzliche Metaanalyse von Arcangeli et al. zeigte eine signifikante Überlegenheit im 5‑Jahres-Überleben mit 57 % (*n* = 3131) vs. 52 % (*n* = 10.265, *p* = 0,04) zugunsten der blasenerhaltenden Radiochemotherapie; dieser Überlebensvorteil blieb auch bestehen, wenn die Zystektomie mit einer neoadjuvanten Chemotherapie kombiniert wurde [8].

Die BC2001-Studie ist für interdisziplinäre Diskussionen wichtig, weil sie die beste Evidenz für die organerhaltende Radiochemotherapie liefert. Weltweit sticht diese britische Studie durch valide Langzeitdaten hervor. Für Radioonkologen sind folgende Aspekte besonders relevant:

- Die simultane Radiochemotherapie kann guten Gewissens in jeder Tumorkonferenz bzw. bei Beratung jedes individuellen Patienten als Therapie der ersten Wahl beim muskelinvasiven Blasenkarzinom empfohlen werden. Es gibt keinen Überlebensnachteil gegenüber der Zystektomie, und 80 % der Patienten können ihre Blase langfristig behalten.
- In den USA und in Deutschland wird zur Strahlensensibilisierung meistens Cisplatin verwendet; neben den prospektiven, nichtrandomisierten Studien (z. B. aus Erlangen) beruht dies vor allem auf einer kleinen kanadischen Studie [9]. Die BC2001-Studie zeigt mit höchster Evidenz, dass mit 5‑FU/MMC eine vermutlich äquieffektive Chemotherapie zur Verfügung steht (mit sogar relativ niedrig dosiertem 5‑FU). Das heißt nicht, dass man auf bewährtes Cisplatin verzichten muss, aber die Option 5‑FU/MMC verbreitert sicher das Indikationsspektrum für die Radiochemotherapie in dem älter werdenden Patientenkollektiv, und wir sollten öfter an diese Option denken.
- Nichtradioonkologen sehen in der Radiochemotherapie, wenn überhaupt, die einzige Alternative zur Zystektomie und empfehlen gelegentlich eine Operation, wenn eine Radiochemotherapie nicht möglich ist. Die BC2001-Studie zeigt ganz klar, dass auch die alleinige Radiotherapie eine ziemlich gute Option ist.
- Die hier durch 2 × 2-Design geprüfte zweite Fragestellung zur Dosiseskalation ergab keine Vorteile. Die bisher üblichen Konzepte (ganze Blase, ca. 60 Gy in konventioneller Fraktionierung) bleiben also Standard.
- Nicht vergessen sollte man auch die Möglichkeit der moderaten Hypofraktionierung. In einer zwischenzeitlichen Metaanalyse mit 782 Patienten in der BC2001- und BCON-Studie (Carbogen und Nicotinamid als Sensitizer) war das hypofraktionierte Schema mit 55 Gy in 20 Fraktionen der Standardfraktionierung bezüglich der invasiven lokoregionären Tumorkontrolle sogar überlegen und in puncto (Spät‑)Toxizität nicht schlechter [10].
- Ob zukünftig eine Immuntherapie in Kombination mit Strahlentherapie eine Rolle spielen wird, ist noch unklar. Das Transitionalzellkarzinom der Blase gilt als immunologisch „heißer“ Tumor. Bisher gibt es allerdings kleine einarmige Phase-II-Studien. Beispielsweise zeigten sich in der RACE-IT-Studie (4 Zyklen Nivolumab 240 mg q2w, Start eine Woche vor Bestrahlungsbeginn, 50,40 Gy auf Blase/Becken, gefolgt von radikaler Zystektomie, keine Chemotherapie) eine radiologische Gesamtansprechrate von 71 % (davon 16 % CR) sowie 39 % pathologische Komplettremission (ypT0) nach anschließender Zystektomie [11]. Die Ergebnisse randomisierter Studien mit Pembrolizumab (Keynote-992) und Durvalumab (RadIO ISRCTN43698103) werden erwartet.

Zusammenfassend zeigt die BC2001-Studie erneut und eindrucksvoll, dass eine trimodale organerhaltende Radiochemotherapie eine valide Alternative zur radikalen Zystektomie darstellt.

*Felix Grabenbauer und Michael Flentje, Würzburg*
